# Supplementary material for: Mycobacterium chimaera Infections in a Unit of Cardio Surgery: Study at a General Hospital in Padua, Italy
Source: Microorganisms. 2023 Dec 23;12(1):29. doi: 10.3390/microorganisms12010029 (PMC10818605; doi:10.3390/microorganisms12010029)
Supplement: Supplementary file 1 [file microorganisms-12-00029-s001.zip › microorganisms-2783900-supplementary.pdf]

Supplementary materials

Table S1. Distribution of the positivities by month in the pre disinfection samples.

Pre	Months	Type 1			Type 2			Total		
		Samples	Positivities		Samples	Positivities		Samples	Positivities	
		N	N	(%)	N	N	(%)	N	N	(%)
2017	Jan-Apr									
	May-Aug									
	Sept-Dec									
2018	Jan-Apr									
	May-Aug									
	Sept-Dec									
2019	Jan									
	Feb									
	Mar									
	Apr									
	May									
	June									
	July	3	2	(66,7)	2	1	(50,0)	5	3	(60,0)
	Aug				2	2	(100,0)	2	2	(100,0)
	Sept	2	2	(100,0)				2	2	(100,0)
	Oct									
	Nov				2	0	(0,0)	2	0	(0,0)
	Dec				1	0	(0,0)	1	0	(0,0)
2020	Jan	5	1	(20,0)	3	0	(0,0)	8	1	(12,5)
	Feb	5	1	(20,0)	16	2	(12,5)	21	3	(14,3)
	Mar	9	2	(22,2)	9	1	(11,1)	18	3	(16,7)

	Apr									
	May									
	June	5	1	(20,0)				5	1	(20,0)
	July	10	3	(30,0)	40	1	(2,5)	50	4	(8,0)
	Aug	12	4	(33,3)	22	0	(0,0)	34	4	(11,8)
	Sept	10	6	(60,0)	30	1	(3,3)	40	7	(17,5)
	Oct	10	4	(40,0)	38	0	(0,0)	48	4	(8,3)
	Nov	8	3	(37,5)	26	0	(0,0)	34	3	(8,8)
	Dec	9	1	(11,1)	22	0	(0,0)	31	1	(3,2)
2021	Jan	5	2	(40,0)	15	0	(0,0)	20	2	(10,0)
	Feb	4	1	(25,0)	11	0	(0,0)	15	1	(6,7)
	Mar	4	1	(25,0)	13	1	(7,7)	17	2	(11,8)
	Apr	5	2	(40,0)	11	0	(0,0)	16	2	(12,5)
	May	5	1	(20,0)	15	0	(0,0)	20	1	(5,0)
	June	4	2	(50,0)	11	0	(0,0)	15	2	(13,3)
	July	4	1	(25,0)	10	0	(0,0)	14	1	(7,1)
	Aug	4	1	(25,0)	5	0	(0,0)	9	1	(11,1)
	Sept	3	1	(33,3)	1	0	(0,0)	4	1	(25,0)
	Oct	3	1	(33,3)	1	0	(0,0)	4	1	(25,0)
	Nov	4	1	(25,0)	5	0	(0,0)	9	1	(11,1)
	Dec	4	0	(0,0)	18	0	(0,0)	22	0	(0,0)
2022	Jan	4	3	(75,0)	4	0	(0,0)	8	3	(37,5)
	Feb	4	2	(50,0)	2	0	(0,0)	6	2	(33,3)
	Mar	4	3	(75,0)	2	0	(0,0)	6	3	(50,0)
	Apr	5	2	(40,0)	2	0	(0,0)	7	2	(28,6)
	May	3	0	(0,0)	8	0	(0,0)	11	0	(0,0)
	**Total**	**157**	**54**	**(34,4)**	**347**	**9**	**(2,6)**	**504**	**63**	**(12,5)**

Table S2. Distribution of positivities by month in the post-disinfection samples.

Post	Month	Type 1			Type 2			Total		
		Samples	Positivities		Samples	Positivities		Samples	Positivities	
		N	N	(%)	N	N	(%)	N	N	(%)
2017	Jan-Apr	6	0	(0,0)	4	0	(0,0)	10	0	(0,0)
	May-Aug									
	Sept-Dec	7	0	(0,0)				7	0	(0,0)
2018	Jan-Apr				7	0	(0,0)	7	0	(0,0)
	May-Aug	5	0	(0,0)				5	0	(0,0)
	Sept-Dec	5	0	(0,0)				5	0	(0,0)
2019	Jan									
	Feb				3	2	(66,7)	3	2	(66,7)
	Mar	6	2	(33,3)	9	2	(22,2)	15	4	(26,7)
	Apr	3	0	(0,0)				3	0	(0,0)
	May									
	June	5	2	(40,0)	12	12	(100,0)	17	14	(82,4)
	July	5	1	(20,0)	16	7	(43,8)	21	8	(38,1)
	Aug	1	0	(0,0)	2	1	(50,0)	3	1	(33,3)
	Sept	4	1	(25,0)				4	1	(25,0)
	Oct									
	Nov				12	0	(0,0)	12	0	(0,0)
	Dec	4	3	(75,0)	2	0	(0,0)	6	3	(50,0)
2020	Jan	11	1	(9,1)	3	0	(0,0)	14	1	(7,1)
	Feb	7	0	(0,0)	16	2	(12,5)	23	2	(8,7)
	Mar	9	0	(0,0)	10	0	(0,0)	19	0	(0,0)
	Apr									
	May									
	June	5	0	(0,0)				5	0	(0,0)
	July	10	1	(10,0)	40	0	(0,0)	50	1	(2,0)

	Aug	12	2	(16,7)	22	0	(0,0)	34	2	(5,9)
	Sept	11	1	(9,1)	30	0	(0,0)	41	1	(2,4)
	Oct	12	2	(16,7)	38	0	(0,0)	50	2	(4,0)
	Nov	14	3	(21,4)	26	0	(0,0)	40	3	(7,5)
	Dec	9	1	(11,1)	22	0	(0,0)	31	1	(3,2)
2021	Jan	5	0	(0,0)	17	0	(0,0)	22	0	(0,0)
	Feb	5	0	(0,0)	11	0	(0,0)	16	0	(0,0)
	Mar	5	0	(0,0)	13	0	(0,0)	18	0	(0,0)
	Apr	6	3	(50,0)	11	0	(0,0)	17	3	(17,6)
	May	6	1	(16,7)	15	0	(0,0)	21	1	(4,8)
	June	5	1	(20,0)	11	0	(0,0)	16	1	(6,3)
	July	4	1	(25,0)	14	0	(0,0)	18	1	(5,6)
	Aug	4	0	(0,0)	5	0	(0,0)	9	0	(0,0)
	Sept	2	1	(50,0)	1	0	(0,0)	3	1	(33,3)
	Oct	4	1	(25,0)	1	0	(0,0)	5	1	(20,0)
	Nov	4	0	(0,0)	5	0	(0,0)	9	0	(0,0)
	Dec	4	0	(0,0)	18	0	(0,0)	22	0	(0,0)
2022	Jan	4	0	(0,0)	4	0	(0,0)	8	0	(0,0)
	Feb	4	0	(0,0)	2	0	(0,0)	6	0	(0,0)
	Mar	5	1	(20,0)	2	0	(0,0)	7	1	(14,3)
	Apr	5	0	(0,0)	2	0	(0,0)	7	0	(0,0)
	May	4	0	(0,0)	8	0	(0,0)	12	0	(0,0)
**Total**		**227**	**29**	**(12,8)**	**414**	**26**	**(6,3)**	**641**	**55**	**(8,6)**
